# Semantic Algorithmic Information Theory: From Kolmogorov Complexity to Semantic Equivalence

**DOI:** 10.3390/e28050554

**Published:** 2026-05-14

**Authors:** Jiatong Wu, Sen Wang, Kai Niu, Yifei She, Ping Zhang

**Affiliations:** 1 State Key Laboratory of Networking and Switching Technology, Beijing University of Posts and Telecommunications, Beijing 100876, China; wujt@bupt.edu.cn (J.W.); pzhang@bupt.edu.cn (P.Z.); 2 China Mobile Research Institute, Beijing 100080, China; wangsenyjy@chinamobile.com; 3 Key Laboratory of Universal Wireless Communications, Ministry of Education, Beijing University of Posts and Telecommunications, Beijing 100876, China; sheyifei@bupt.edu.cn

**Keywords:** algorithmic information theory, kolmogorov complexity, semantic information theory, normalized semantic information distance, synonymous set, set-theoretic metrics

## Abstract

Classical Algorithmic Information Theory (AIT) provides a rigorous foundation for information-based similarity measurement, but classical formulations and their compression-based approximations largely operate at the syntactic level, making them sensitive to surface-level variation and insufficient for semantic equivalence. To address this limitation, this paper introduces Semantic Algorithmic Information Theory. The contributions are organized around three core aspects. First, regarding algorithmic extension, we formalize the Semantic Turing Machine System (STMS) to decouple abstract concepts from their diverse syntactic realizations. Within this framework, Semantic Complexity is defined as the minimum program length required to generate some realization in a synonymous set, thereby characterizing compact meaning representation. Second, to enable approximate computation, we move from the ideal, uncomputable semantic information distance to a model-based direct estimator of the Normalized Semantic Information Distance (NSID), which uses neural autoregressive models as conditional probability estimators. Finally, through experimental validation and comparative analysis, we show that the NSID estimator suppresses syntactic variance while preserving semantic structure. Empirical results indicate that NSID provides a practical, computable surrogate for semantic distance and improves upon classical syntactic metrics in evaluating cross-representational equivalence.

## 1. Introduction

The fundamental goal of information theory is to quantify the amount of information contained in data and the information shared between different objects. While Shannon’s classical information theory [1] established the statistical limits of communication, it deliberately abstracted away the “meaning” of messages. To measure the absolute information content of individual objects, Algorithmic Information Theory (AIT) introduced Kolmogorov complexity [2], formally defined as the length of the shortest program required to compute a string on a universal Turing machine. Beyond mere compression, Kolmogorov’s framework also provides the ultimate basis for model selection and determining the structural properties of data [3]. Building on this, the Information Distance [4] and its normalized form, the Normalized Information Distance (NID) [5], were developed.

Approximated in practice via the Normalized Compression Distance (NCD) [6], this framework has served as a principled baseline for parameter-free similarity measurement, with extensions and applications ranging from multiset similarity [7] to image similarity detection [8] and low-resource text classification [9].

However, a fundamental limitation persists: classical AIT and practical NCD remain representation-dependent and largely operate at the syntactic level. Standard Turing machines and classical compression algorithms operate on surface-level bytes or tokens. They evaluate the algorithmic cost of transforming one exact string into another. Consequently, traditional metrics are highly sensitive to “syntactic noise.” For instance, an English sentence and its precise French translation share almost identical semantic information. Yet, classical NCD would report a large distance between them due to completely different vocabularies and syntactic structures.

Recently, the success of Large Language Models (LLMs) has renewed the connection between prediction, compression, and understanding. The “Language Modeling is Compression” paradigm [10] and subsequent large-scale compression results [11] show that neural models can serve as strong lossless compressors. However, a critical paradox remains: replacing classical compressors with LLM-based neural compressors in NCD does not consistently improve classification accuracy and can even underperform traditional compressors despite better compression rates [12]. This suggests that stronger probabilistic compression alone is insufficient for semantic similarity, motivating an information-distance formulation in semantic space.

Although advanced representation learning and embedding models, such as EmbeddingGemma [13], have achieved strong empirical performance in semantic tasks, their connection to the algorithmic information bounds of Kolmogorov complexity remains underdeveloped. Related work has incorporated semantic similarity into predictability-oriented information measures [14,15], but these approaches remain closer to probabilistic predictability than to algorithmic information distance. In parallel, semantic communication theory [16,17] has generalized Shannon’s framework through synonymous mappings, semantic entropy, and semantic channel capacity, but does not directly provide a metric space for individual algorithmic objects.

To bridge this theoretical gap and resolve the LLM-NCD paradox, this paper introduces Semantic Algorithmic Information Theory. We explicitly decouple the complexity of abstract concepts from the complexity of their syntactic realizations. Our core theoretical contributions begin with the definition of the Semantic Turing Machine System (STMS), which integrates a classical Turing machine with a computable synonymous mapping. Under this model, we formalize Semantic Complexity, establishing a rigorous boundary between semantic entropy and semantic algorithmic complexity.

Furthermore, to transition from abstract theory to practical measurement, we propose a model-based direct approximation to the **Normalized Semantic Information Distance (NSID)**. Rather than blindly applying the NCD formula—which has proven problematic with LLMs [12]—NSID is a conceptual reformulation of the ideal NID specifically tailored for the semantic space. By utilizing LLMs as conditional probability estimators, the resulting Direct NSID estimator computes a model-induced informational cost of generating a target sequence given a semantic context. Because observable systems inevitably introduce a syntactic transformation cost, point-wise comparisons are insufficient. Therefore, we establish a set-theoretic framework—utilizing Intra-Synonymous Set Cohesion, Inter-Synonymous Set Separability, and the Discrimination Ratio—to statistically quantify the phenomenon of Semantic Equivalence: the state where a system substantially suppresses syntactic noise and keeps diverse synonymous realizations computationally close.

The rest of the paper is organized as follows: Section 2 recalls necessary background on Kolmogorov complexity and semantic entropy. Section 3 introduces the Semantic Turing Machine and Semantic Complexity with formal definitions and proofs. Section 4 establishes the ideal semantic metric space and the set-theoretic framework. Section 5 defines the computable NSID estimator via LLMs. Extensive empirical validations demonstrating cross-lingual semantic equivalence are presented in Section 6, followed by the conclusion.

## 2. Preliminaries

### 2.1. Kolmogorov Complexity and Information Distance

We first recall some basic notation and facts regarding Kolmogorov complexity found in standard texts [2]. Let ${\left\{0,1\right\}}^{*}$ denote the set of all finite binary strings. The Kolmogorov complexity, or algorithmic entropy, K(x) of a string *x* is formally defined as the length of the shortest binary program *p* that, when executed on a fixed universal Turing machine $U_{0}$, computes *x* and halts; i.e., $K\left(x\right)=\min\{|p|:U_{0}\left(p\right)=x\}$. Essentially, K(x) represents the length of the ultimate compressed version of *x*. The conditional Kolmogorov complexity K(x∣y) is defined similarly as the length of the shortest program to compute *x* when *y* is provided as an auxiliary input. Although defined relative to the specific machine $U_{0}$, the Invariance Theorem ensures that these values are machine-independent up to an additive constant, endowing K(x) with an absolute character.

Based on these concepts, Bennett et al. [4] defined the information distance E(x,y) between two strings *x* and *y* as the length of the shortest binary program that transforms *x* into *y* as well as *y* into *x*. Such a program functions in a catalytic capacity: it facilitates the transformation between inputs while remaining present and unchanged throughout the computation. It was proven that this distance is equal to the maximum of the conditional complexities, up to a logarithmic additive term:

(1) E(x,y)=max{K(x∣y),K(y∣x)}+O(logmax{K(x),K(y)}).

To enable comparisons between sequences of different lengths, Li et al. [5] introduced the Normalized Information Distance (NID). The NID normalizes the information distance by the maximum complexity of the two strings, yielding a universal metric that is asymptotically bounded in [0,1] up to the standard additive terms.

(2) $$\mathrm{NID}\left(x,y\right)=\frac{\max\{K(x|y),K(y|x)\}}{\max\{K(x),K(y)\}}.$$

The NID is considered the “gold standard” for similarity metrics because it minorizes all other computable normalized metrics; a lower NID indicates that *x* and *y* share more information.

Since the function K(·) is non-computable, the NID cannot be directly calculated. In practice, we approximate K(x) using a standard real-world compression algorithm *C*, where C(x) denotes the length of the compressed string *x*. This leads to the Normalized Compression Distance (NCD), a computable approximation of the NID defined as follows:

(3) $$\mathrm{NCD}\left(x,y\right)=\frac{C(xy)-\min\{C(x),C(y)\}}{\max\{C(x),C(y)\}},$$

where xy represents the concatenation of strings *x* and *y*. The term C(xy) captures the shared information between the two strings, relying on the compressor’s ability to exploit redundancy in the concatenated sequence.

### 2.2. Semantic Entropy and Synonymous Mapping

Following the framework of semantic information theory [16,17], we consider a discrete syntactic symbol set $U=\{u_{1},\ldots ,u_{N}\}$ and a corresponding semantic symbol set $\tilde{U}=\left\{{\tilde{u}}_{1},\ldots ,{\tilde{u}}_{\tilde{N}}\right\}$. A synonymous mapping $f:U\to \tilde{U}$ establishes the correspondence between these spaces by mapping syntactically distinct symbols to their semantic equivalents.

This mapping induces a partition of the syntactic space U into disjoint synonymous sets (or equivalence classes). For each semantic entity ${\tilde{u}}_{k}\in \tilde{U}$, the corresponding synonymous set $U_{k}$ is defined as the set of all syntactic symbols that map to ${\tilde{u}}_{k}$:

(4) $$U_{k}=\left\{u\in U:f\left(u\right)={\tilde{u}}_{k}\right\}.$$

These sets satisfy the properties of a partition: $U_{k}\cap U_{j}=\emptyset$ for k≠j, and ${⋃}_{k}U_{k}=U$.

Let *U* be a discrete random variable taking values in U with probability mass function p(u). We define the semantic variable $\tilde{U}=f\left(U\right)$ as the random variable associated with the semantic entities. The probability distribution of $\tilde{U}$ is derived from *U* by aggregating the probabilities of the syntactic symbols within each synonymous set:

(5) $$p\left({\tilde{u}}_{k}\right)≜p\left(U_{k}\right)=\sum_{u\in U_{k}}p\left(u\right).$$

The semantic entropy $H_{s}\left(\tilde{U}\right)$ is formally defined as the Shannon entropy of the semantic variable $\tilde{U}$. It quantifies the uncertainty associated with the semantic content:

(6) $$H_{s}\left(\tilde{U}\right)≜-\sum_{k=1}^{\tilde{N}}p\left({\tilde{u}}_{k}\right)\log p\left({\tilde{u}}_{k}\right)=-\sum_{k=1}^{\tilde{N}}\left(\sum_{u\in U_{k}}p\left(u\right)\right)\log\left(\sum_{u\in U_{k}}p\left(u\right)\right).$$

A fundamental property of semantic entropy is that it is upper-bounded by the syntactic entropy: $H_{s}\left(\tilde{U}\right)=H\left(f\left(U\right)\right)\leq H\left(U\right)$. The inequality is strict when *f* merges at least two syntactic symbols with positive probability; otherwise equality may hold.

## 3. Theory of Semantic Algorithmic Information

In this section, we formally extend Algorithmic Information Theory (AIT) to the semantic domain. We establish the computational model, define semantic complexity metrics, and prove fundamental theorems regarding universality, invariance, and semantic reduction.

### 3.1. Semantic Turing Machine System

To define complexity relative to semantics, we must extend the classical model of computation to incorporate semantic processing.

We use the same symbol *f* for the computable synonymous mapping $f:{\left\{0,1\right\}}^{*}\to \tilde{U}$, as the algorithmic counterpart of the entropy-theoretic mapping $f:U\to \tilde{U}$ introduced above.

**Definition** **1** (Semantic Turing Machine System). *A Semantic Turing Machine System (STMS) is a pair (T,f), where*

*1.*  *T is a standard prefix Turing machine (with a unidirectional input tape, a working tape, and a unidirectional output tape).*
*2.*  $f:{\left\{0,1\right\}}^{*}\to \tilde{U}$ is a computable surjective function, termed the synonymous mapping, which maps syntactic strings to their semantic representations in a semantic universe $\tilde{U}$.

For any binary program *p*, the operation of the system is defined as follows: First, *T* executes program *p*. If *T* halts and outputs a string $z\in {\left\{0,1\right\}}^{*}$, the system then applies *f* to *z*. The final semantic output is $\tilde{u}=f\left(z\right)$. We say that program *p* semantically generates *x* if f(T(p))=f(x).

**Definition** **2** (Synonymous Mapping and Synonymous Set). Let $f:{\left\{0,1\right\}}^{*}\to \tilde{U}$ be a synonymous mapping that maps syntactically different but semantically equivalent strings to the same semantic entity $\tilde{u}$. This function defines the relationship between syntax (binary strings) and their corresponding semantics.

For any string $x\in {\left\{0,1\right\}}^{*}$, we define the synonymous set of x under f as follows:

(7) $${\left[x\right]}_{f}:=\left\{z\in {\left\{0,1\right\}}^{*}:f\left(z\right)=f\left(x\right)\right\}.$$

*Thus, ${\left[x\right]}_{f}$ collects all syntactic realizations semantically equivalent to x, allowing semantic complexity to ignore surface-form variation.*

**Figure 1:**
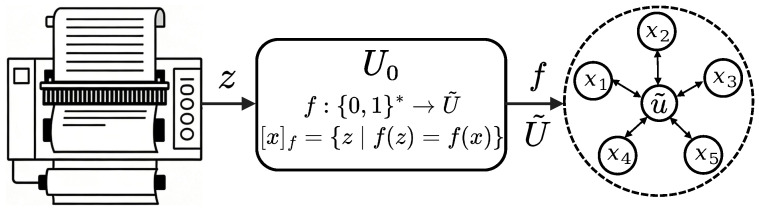
Architecture of the universal Semantic Turing Machine System $\left(U_{0},f\right)$.

To provide a more intuitive understanding, Figure 1 illustrates the architecture of the STMS. As shown on the left, the universal reference machine $U_{0}$ executes a program to generate a syntactic string *z*. The central mapping function *f* then transforms this string into a semantic entity $\tilde{u}$. On the right, the dashed boundary represents the synonymous set ${\left[x\right]}_{f}$, visually illustrating how multiple distinct syntactic strings (e.g., $x_{1},\ldots ,x_{5}$) converge onto the same underlying semantic concept. This decoupling of syntax and semantics is essential for defining complexity based on meaning rather than surface form.

**Definition** **3** (Admissible Synonymous Mapping). *To avoid trivial solutions (e.g., mapping all strings to a constant), a mapping f is admissible if it satisfies the non-degeneracy condition. Formally, for the source distribution under consideration, f is admissible if the induced semantic random variable f(X) has at least two semantic outcomes with positive probability, equivalently H(f(X))>0.*

### 3.2. Universality and Invariance

A core requirement for any algorithmic complexity measure is invariance with respect to the underlying computational model.

For an arbitrary STMS (A,f), define

(8) $$K_{f,A}\left(x\right):=\min\left\{|p|:f\left(A\left(p\right)\right)=f\left(x\right)\right\}.$$

When $A=U_{0}$, this becomes $K_{f,U_{0}}\left(x\right)$.

**Theorem** **1** (Universality of Semantic Systems). *Let $U_{0}$ be a universal prefix Turing machine. Then $\left(U_{0},f\right)$ constitutes a universal STMS. For any other STMS (A,f) based on a machine A, there exists a constant $c_{A}$ (dependent on A but independent of the semantic target) such that for all x:*

(9) $$K_{f,U_{0}}\left(x\right)\leq K_{f,A}\left(x\right)+c_{A}.$$

**Proof.**  Let $s_{A}$ be the simulation prefix for machine *A* such that $U_{0}\left(s_{A}p\right)=A\left(p\right)$ for all programs *p*. Let $p^{*}$ be the shortest program satisfying $f\left(A\left(p^{*}\right)\right)=f\left(x\right)$, so that $\left|p^{*}\right|=K_{f,A}(x)$. The concatenated program $p^{\prime }=s_{A}p^{*}$ simulates *A* on $U_{0}$, yielding a syntactic output *z* with f(z)=f(x). Thus, $p^{\prime }$ semantically generates *x* on $\left(U_{0},f\right)$ and has length $\left|p^{\prime }\right|=\left|s_{A}\right|+\left|p^{*}\right|=c_{A}+K_{f,A}\left(x\right)$.    □

In the following, we fix the universal reference system $\left(U_{0},f\right)$ and write $K_{f}\left(x\right)$ for $K_{f,U_{0}}\left(x\right)$.

### 3.3. Semantic Complexity

We distinguish between the complexity of a syntactic string relative to its meaning ($K_{f}\left(x\right)$) and the complexity of the abstract concept itself ($K_{s}\left(\tilde{u}\right)$).

**Definition** **4** (Semantic Complexity). *The semantic complexity of a string x is defined as the minimum description length required to compute its semantic representation:*

(10) $$K_{f}\left(x\right):=\min\{|p|:f\left(U_{0}\left(p\right)\right)=f\left(x\right)\}.$$

We now relate semantic complexity to classical syntactic complexity.

**Theorem** **2** (Syntactic–Semantic Decomposition). *For any string x, the syntactic complexity admits the following decomposition:*

(11) $$K\left(x\right)\leq K_{f}\left(x\right)+K\left(x|p_{f}^{*}\left(x\right)\right)+O\left(\log K\left(x\right)\right),$$

where $p_{f}^{*}\left(x\right)$ denotes a shortest program satisfying $f(U_{0}\left(p_{f}^{*}\left(x\right)\right))=f\left(x\right)$.

**Proof.**  The result follows from the chain rule of Kolmogorov complexity. A description of *x* can be given by a two-part, self-delimiting code: first a program that generates its semantic content, and second a description that specifies the particular syntactic realization among all strings sharing that semantic value. The logarithmic term accounts for the overhead of delimiting and combining the two parts.    □

**Corollary** **1** (Semantic Reduction). *For any string x, semantic complexity is equal, up to an additive constant, to the minimum syntactic complexity among all strings in its synonymous set:*

(12) $$K_{f}\left(x\right)=\min_{z\in {\left[x\right]}_{f}}K\left(z\right)+O\left(1\right).$$

*Consequently, since $x\in {\left[x\right]}_{f}$, semantic complexity is upper-bounded by syntactic complexity:*

(13) $$K_{f}\left(x\right)\leq K\left(x\right)+O\left(1\right).$$

*The gap*

(14) $$\Delta K_{f}\left(x\right)=K\left(x\right)-K_{f}\left(x\right)$$

*quantifies syntactic redundancy, i.e., the information required to specify the particular surface realization once the semantic equivalence class is fixed.*

**Theorem** **3** (Exact Invariance under Synonymous Transformation). Let $\phi :{\left\{0,1\right\}}^{*}\to {\left\{0,1\right\}}^{*}$ be any transformation satisfying

(15) f(ϕ(x))=f(x).

*Then*

(16) $$K_{f}\left(\phi \left(x\right)\right)=K_{f}\left(x\right).$$

**Proof.**  Since f(ϕ(x))=f(x), the set of programs that semantically generate ϕ(x) is identical to the set of programs that semantically generate *x*. Taking the minimum program length over the same set gives $K_{f}\left(\phi \left(x\right)\right)=K_{f}\left(x\right)$.    □

#### Concept-Level Semantic Complexity

The next definition lifts semantic complexity from a string realization to an abstract semantic concept.

**Definition** **5** (Semantic Complexity of Concepts). *For a semantic concept $\tilde{u}\in \tilde{U}$, the unconditional semantic complexity is defined as*

(17) $$K_{s}\left(\tilde{u}\right):=\min\{|p|:f\left(U_{0}\left(p\right)\right)=\tilde{u}\}.$$

For any $x\in {\left\{0,1\right\}}^{*}$, the string-indexed and concept-indexed definitions agree:

(18) $$K_{f}\left(x\right)=K_{s}\left(f\left(x\right)\right).$$

We use $K_{f}\left(x\right)$ for observed syntactic realizations and $K_{s}\left(\tilde{u}\right)$ for concept-level distances.

**Definition** **6** (Conditional Semantic Complexity of Concepts). Let $e:\tilde{U}\to {\left\{0,1\right\}}^{*}$ be a fixed effective encoding of semantic concepts. For two concepts $\tilde{u},\tilde{v}\in \tilde{U}$, the conditional semantic complexity is defined as

(19) $$K_{s}\left(\tilde{u}|\tilde{v}\right):=\min\{|p|:f\left(U_{0}\left(p,e\left(\tilde{v}\right)\right)\right)=\tilde{u}\}.$$

## 4. Semantic Distance and Set-Theoretic Metrics

In the previous section, we established the Semantic Turing Machine framework. To quantitatively evaluate the capability of a system to maintain semantic invariance across different syntactic realizations, we must transition from abstract semantic concepts to a rigorous distance-based framework. In this section, we first define the ideal semantic information distance and its normalized form, then discuss the theoretical inevitability of representation noise, and finally derive a set-theoretic framework for measuring semantic alignment.

### 4.1. The Ideal Semantic Metric Space

Let $\tilde{U}$ denote the semantic universe induced by the synonymous mapping $f:{\left\{0,1\right\}}^{*}\to \tilde{U}$. For each syntactic realization *x*, its associated semantic concept is given by $\tilde{u}=f\left(x\right)$, and the corresponding synonymous set is ${\left[x\right]}_{f}=\left\{z\in {\left\{0,1\right\}}^{*}:f\left(z\right)=f\left(x\right)\right\}$. To analyze the geometry of meaning, we define distances directly on semantic concepts in $\tilde{U}$, rather than on surface-level syntactic strings. Following the classical information distance framework, we first define an ideal semantic information distance and then introduce its normalized form.

**Definition** **7** (Ideal Semantic Information Distance). *Let $\tilde{u},\tilde{v}\in \tilde{U}$ be two abstract semantic concepts. The ideal semantic information distance is defined as*

(20) $$E_{s}\left(\tilde{u},\tilde{v}\right)=\max\left\{K_{s}\left(\tilde{u}|\tilde{v}\right),K_{s}\left(\tilde{v}|\tilde{u}\right)\right\}.$$

*Here $K_{s}\left(\cdot \right)$ and $K_{s}\left(\cdot |\cdot \right)$ denote the concept complexity and conditional concept complexity defined in Definitions 5 and 6.*

The distance $E_{s}$ is the semantic analogue of the classical information distance. In the algorithmic setting, it is interpreted up to the standard additive terms of Kolmogorov complexity. In this ideal sense, it satisfies the following metric-like properties for any $\tilde{u},\tilde{v},\tilde{w}\in \tilde{U}$:

1. **Non-negativity:** $E_{s}\left(\tilde{u},\tilde{v}\right)\geq 0$.
2. **Identity of Indiscernibles:** $E_{s}\left(\tilde{u},\tilde{v}\right)=O\left(1\right)$ if $\tilde{u}=\tilde{v}$, and is positive up to additive constants when $\tilde{u}\neq \tilde{v}$.
3. **Symmetry:** $E_{s}\left(\tilde{u},\tilde{v}\right)=E_{s}\left(\tilde{v},\tilde{u}\right)$.
4. **Triangle Inequality:** $E_{s}\left(\tilde{u},\tilde{w}\right)\leq E_{s}\left(\tilde{u},\tilde{v}\right)+E_{s}\left(\tilde{v},\tilde{w}\right)+O\;\left(\log\max\{K_{s}\left(\tilde{u}\right),K_{s}\left(\tilde{v}\right),K_{s}\left(\tilde{w}\right)\}\right).$

**Definition** **8** (Ideal Normalized Semantic Information Distance). *The ideal Normalized Semantic Information Distance (NSID) is defined as the normalized form of $E_{s}$:*

(21) $${\mathrm{NSID}}_{s}\left(\tilde{u},\tilde{v}\right)=\frac{E_{s}\left(\tilde{u},\tilde{v}\right)}{\max\{K_{s}\left(\tilde{u}\right),K_{s}\left(\tilde{v}\right)\}}.$$

*Equivalently,*

(22) $${\mathrm{NSID}}_{s}\left(\tilde{u},\tilde{v}\right)=\frac{\max\{K_{s}\left(\tilde{u}|\tilde{v}\right),K_{s}\left(\tilde{v}|\tilde{u}\right)\}}{\max\{K_{s}\left(\tilde{u}\right),K_{s}\left(\tilde{v}\right)\}}.$$

*In the ideal normalized setting, ${\mathrm{NSID}}_{s}$ is bounded by 1 up to the usual additive and normalization terms of algorithmic information theory. It provides the normalized semantic distance used in the observation model and in the empirical estimators below.*

### 4.2. Theoretical Observation Model and System Noise

While ${\mathrm{NSID}}_{s}$ defines the ideal normalized distance between abstract semantic concepts, external observers (and computational systems) can only measure distances between syntactic realizations $x,y\in {\left\{0,1\right\}}^{*}$. Even with perfect synonymous mapping, the variation in surface form introduces an inherent cost.

We model the Observed Empirical Distance $\hat{d}\left(x,y\right)$ as a noisy observation of the ideal normalized semantic distance under the following additive observation hypothesis.

**Definition** **9** (Observed Empirical Distance). *For any two syntactic realizations x,y, the observed distance is decomposed as follows:*

(23) $$\hat{d}\left(x,y\right)={\mathrm{NSID}}_{s}\left(f\left(x\right),f\left(y\right)\right)+\epsilon_{\mathrm{sys}}\left(x,y\right),$$

*where $\epsilon_{\mathrm{sys}}\left(x,y\right)\geq 0$ represents the Syntactic Transformation Cost.*

**Remark** **1.** *The term $\epsilon_{\mathrm{sys}}$ is not measurement error, but the residual cost of aligning different surface realizations. Thus, even when x and y are synonymous and ${\mathrm{NSID}}_{s}\left(f\left(x\right),f\left(y\right)\right)$ vanishes up to the usual additive and normalization terms, the observed distance may remain positive, which motivates the set-theoretic metrics below.*

### 4.3. Set-Theoretic Metrics for Semantic Topology

Given the existence of $\epsilon_{\mathrm{sys}}$, we cannot rely on single pairs of samples to evaluate semantic integrity. Instead, we analyze the statistical properties of the Synonymous Set $S_{\tilde{u}}=\left\{x_{1},\ldots ,x_{n}\right\}$, which acts as the empirical instantiation under the synonymous mapping *f*, containing *n* distinct syntactic realizations of a single semantic concept $\tilde{u}$.

#### 4.3.1. Intra-Synonymous Set Cohesion (Semantic Compactness)

The first requirement for a robust semantic system is that realizations of the same meaning should form a compact cluster. We quantify this by the average pairwise distance within a synonymous set.

**Definition** **10** (Intra-Synonymous Set Cohesion).

(24) $$C_{\mathrm{intra}}\left(S_{\tilde{u}}\right)=\frac{1}{n(n-1)}\sum_{x\in S_{\tilde{u}}}\sum_{y\in S_{\tilde{u}},y\neq x}\hat{d}\left(x,y\right)$$

For within-set pairs ($x,y\in S_{\tilde{u}}$), this metric estimates the expected syntactic transformation cost $E[\epsilon_{\mathrm{sys}}]$ for concept $\tilde{u}$. A lower $C_{\mathrm{intra}}$ indicates high semantic invariance, where the system treats different phrasings as functionally similar.

#### 4.3.2. Inter-Synonymous Set Separability (Semantic Divergence)

Distinct concepts must be topologically separable. For two disjoint synonymous sets $S_{\tilde{u}}$ and $S_{\tilde{v}}$ (where $\tilde{u}\neq \tilde{v}$), separability is the average distance between their elements.

**Definition** **11** (Inter-Synonymous Set Separability). *Let $|S_{\tilde{u}}|=n$ and $|S_{\tilde{v}}|=m$.*

(25) $$D_{\mathrm{inter}}\left(S_{\tilde{u}},S_{\tilde{v}}\right)=\frac{1}{n\cdot m}\sum_{x\in S_{\tilde{u}}}\sum_{y\in S_{\tilde{v}}}\hat{d}\left(x,y\right)$$

#### 4.3.3. Semantic Discrimination Ratio

To define the boundary between effective alignment and metric failure, we propose the Discrimination Ratio, representing the signal-to-noise ratio of the semantic space.

**Definition** **12** (Semantic Discrimination Ratio).

(26) $$R_{\mathrm{disc}}\left(S_{\tilde{u}},S_{\tilde{v}}\right)=\frac{D_{\mathrm{inter}}\left(S_{\tilde{u}},S_{\tilde{v}}\right)}{\frac{1}{2}\left(C_{\mathrm{intra}}\left(S_{\tilde{u}}\right)+C_{\mathrm{intra}}\left(S_{\tilde{v}}\right)\right)}$$

**Definition** **13** (Condition of Semantic Indistinguishability). *A system is in a state of Semantic Indistinguishability (Topological Degeneracy) with respect to concepts $\tilde{u}$ and $\tilde{v}$ if*

(27) $$R_{\mathrm{disc}}\left(S_{\tilde{u}},S_{\tilde{v}}\right)\leq 1+\delta$$

*where δ is a small margin of error. This condition implies that the semantic distance between distinct concepts has become statistically indistinguishable from the syntactic noise within a single concept, indicating a failure to discriminate distinct semantic concepts.*

## 5. Practical Approximation: Model-Based Direct NSID

In the previous section, we defined the ideal Normalized Semantic Information Distance ${\mathrm{NSID}}_{s}$ on abstract semantic concepts. Since this quantity depends on semantic Kolmogorov complexities, it is not directly computable. In this section, we introduce a model-based direct estimator of NSID, denoted by ${\hat{\mathrm{NSID}}}_{M}$, using Large Language Models (LLMs) as conditional probability estimators. We then substitute this estimator into the set-theoretic formulas to obtain the concrete computational methods used in our analysis.

### 5.1. From Complexity to Conditional Probability

The core challenge is to approximate conditional semantic concept complexity, such as $K_{s}\left(\tilde{u}|\tilde{v}\right)$, from observable syntactic sequences. Classical NCD relies on joint compression terms such as C(xy), whereas neural conditional sequence models allow conditional code lengths to be estimated directly through sequence likelihoods. Let M be a pre-trained conditional sequence model, including decoder-only autoregressive and encoder–decoder architectures, and let $\tau_{M}$ denote its tokenizer. For a syntactic realization *x*, write $\tau_{M}\left(x\right)=\left(x_{1},\ldots ,x_{L_{x}}\right)$ for the token sequence induced by M. Let ${\mathrm{ctx}}_{M}\left(y\right)$ denote the model-specific conditioning input induced by *y*. The conditional code length of *x* is defined by the Shannon–Fano code length induced by the model likelihood:

(28) $$C_{M}\left(x|y\right)=-\log_{2}P_{M}\left(\tau_{M}\left(x\right)|{\mathrm{ctx}}_{M}\left(y\right)\right)=-\sum_{t=1}^{L_{x}}\log_{2}P_{M}\left(x_{t}|x_{<t},{\mathrm{ctx}}_{M}\left(y\right)\right).$$

When no conditioning context is provided, $C_{M}\left(x\right)$ denotes the unconditional code length of *x*:

(29) $$C_{M}\left(x\right)=-\sum_{t=1}^{L_{x}}\log_{2}P_{M}\left(x_{t}|x_{<t}\right).$$

### 5.2. The Direct NSID Estimator

Based on the conditional code length, we define the computable implementation of the empirical distance $\hat{d}\left(x,y\right)$. We term this model-based estimator the Direct Normalized Semantic Information Distance.

**Definition** **14** (Direct NSID Estimator). *The Direct NSID estimator between two syntactic realizations x and y is defined as follows:*

(30) $${\hat{\mathrm{NSID}}}_{M}\left(x,y\right)=\frac{\max\{C_{M}\left(x|y\right),C_{M}\left(y|x\right)\}}{\max\{C_{M}\left(x\right),C_{M}\left(y\right)\}}.$$

Here, the maximum symmetrizes the two conditional directions to reduce autoregressive directional bias, while the denominator normalizes for sequence length.

### 5.3. Discrete Estimators for Set Metrics

To obtain the numerical results, we substitute the generic $\hat{d}\left(x,y\right)$ from Section 4.3 with the computed ${\hat{\mathrm{NSID}}}_{M}\left(x,y\right)$. Let $S_{\tilde{u}}$ and $S_{\tilde{v}}$ be two finite sets of synonymous realizations with cardinalities $\left|S_{\tilde{u}}\right|=n$ and $\left|S_{\tilde{v}}\right|=m$. The final computational formulas are defined as follows:

#### 5.3.1. Estimator for Intra-Synonymous Set Cohesion


(31) $${\hat{C}}_{\mathrm{intra}}\left(S_{\tilde{u}}\right)=\frac{1}{n(n-1)}\sum_{x\in S_{\tilde{u}}}\sum_{y\in S_{\tilde{u}},y\neq x}{\hat{\mathrm{NSID}}}_{M}\left(x,y\right)$$

#### 5.3.2. Estimator for Inter-Synonymous Set Separability


(32) $${\hat{D}}_{\mathrm{inter}}\left(S_{\tilde{u}},S_{\tilde{v}}\right)=\frac{1}{n\cdot m}\sum_{x\in S_{\tilde{u}}}\sum_{y\in S_{\tilde{v}}}{\hat{\mathrm{NSID}}}_{M}\left(x,y\right)$$

#### 5.3.3. Estimator for Semantic Discrimination Ratio


(33) $${\hat{R}}_{\mathrm{disc}}\left(S_{\tilde{u}},S_{\tilde{v}}\right)=\frac{{\hat{D}}_{\mathrm{inter}}\left(S_{\tilde{u}},S_{\tilde{v}}\right)}{\frac{1}{2}\left({\hat{C}}_{\mathrm{intra}}\left(S_{\tilde{u}}\right)+{\hat{C}}_{\mathrm{intra}}\left(S_{\tilde{v}}\right)\right)}$$

Together, these discrete estimators are used in the experiments below.

## 6. Empirical Validation

To validate the proposed Semantic Algorithmic Information Theory, we conducted a controlled topological analysis across 50 cross-lingual sentences. This empirical validation focuses on **Cross-Lingual Semantic Equivalence**, designed to demonstrate the system’s capacity to significantly reduce syntactic variance and to quantitatively measure Semantic Friction across different languages, thereby providing robust empirical grounding for our theoretical framework.

### 6.1. Experimental Setup

We utilized the FLORES-200 dataset [18] to construct synonymous sets across N=10 distinct semantic concepts. Formally, the *i*-th experimental synonymous set is denoted by $S_{i}:=S_{{\tilde{u}}_{i}}^{\left(5\right)}=\left\{x_{i,\mathrm{en}},x_{i,\mathrm{fr}},x_{i,\mathrm{de}},x_{i,\mathrm{nl}},x_{i,\mathrm{it}}\right\}\subseteq f^{-1}\left({\tilde{u}}_{i}\right)$, where i=0,…,9. Here, $S_{0},\ldots ,S_{9}$ are used as experimental labels for the ten semantic concepts. For each concept, we retrieved its realization in five languages from Germanic and Romance branches: English (en), French (fr), German (de), Dutch (nl), and Italian (it). To establish a rigorous comparison, we evaluated both a syntactic baseline and multiple neural implementations of the semantic method:

- **Syntactic Baseline:** Normalized Compression Distance (NCD) using standard Gzip compression.
- **Semantic Method (Ours):** The model-based Direct NSID estimator computed using two pretrained language models: a T5-based encoder–decoder architecture (google/t5gemma-2-1b-1b, hereafter T5-1B) and a decoder-only large language model (Qwen3-14B, hereafter Qwen3-14B).

**Figure 2:**
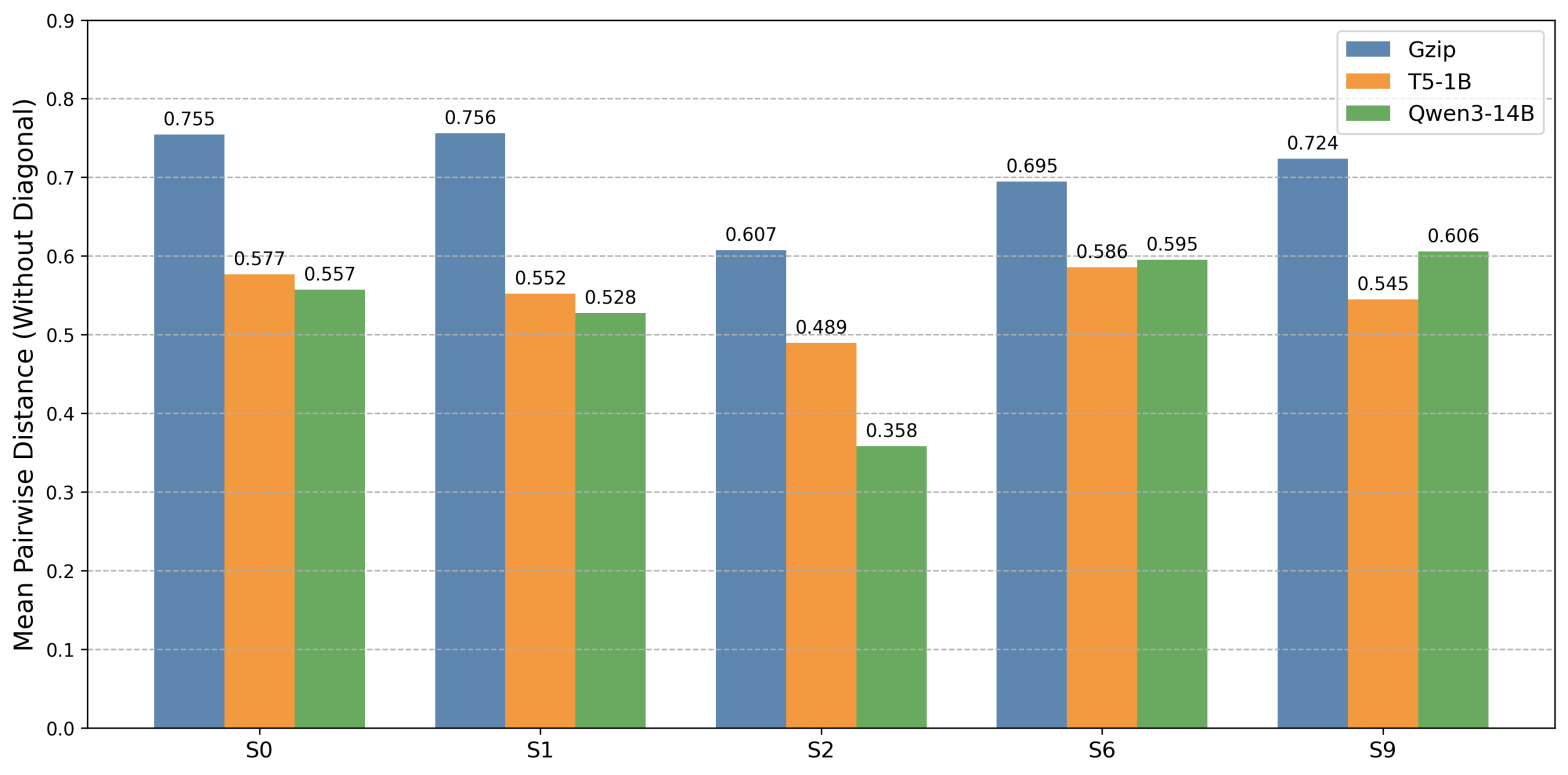
Mean cross-lingual distance within selected synonymous sets.

*A. Model Comparison and Intra-Synonymous Set Cohesion:* Before presenting the full matrix topology, we first compare the mean pairwise distance within five representative cross-lingual synonymous sets, with the full set-level breakdown reported in Table 1. Figure 2 shows that both neural semantic models substantially outperform the classical syntactic baseline. T5-1B and Qwen3-14B achieve broadly comparable intra-set cohesion overall, although each has advantages on individual sets. Given this comparable performance, we adopt T5-1B as the default semantic model in the subsequent experiments because it requires substantially less computation than Qwen3-14B. For readability, we refer to ${\hat{\mathrm{NSID}}}_{M}$ instantiated with T5-1B as T5-NSID in the following analysis. For a concrete multilingual realization format, Appendix A presents the full five-language synonymous set for S6.

**Figure 3:**
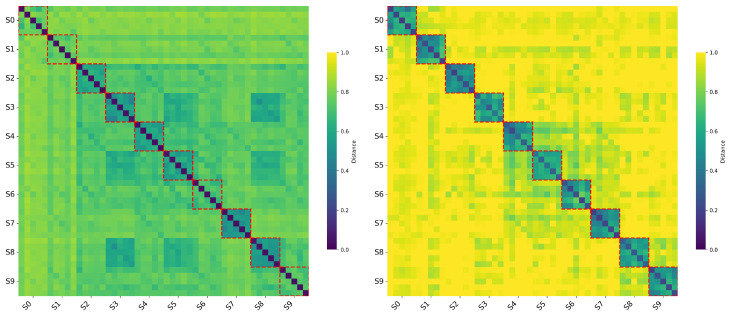
Pairwise distance matrices for semantic equivalence (**left**: Gzip; **right**: T5-1B-based semantic method). The red dashed boxes mark the within-set regions corresponding to the ten cross-lingual synonymous sets, where different-language expressions share the same underlying meaning.

*B. Matrix Topology and Inter-Synonymous Set Separability:*Figure 3 visualizes the full pairwise distance matrices. The baseline (Gzip) exhibits an irregular, diffuse pattern with distances hovering around 0.6 to 0.8 (${\hat{\bar{R}}}_{\mathrm{disc}}\approx 1.20$). Under a practical tolerance δ=0.2, this corresponds to the degeneracy boundary in Definition 13. Because standard compression algorithms rely heavily on character-level redundancy, they are dominated by surface-level syntactic complexity and fail to capture cross-lingual semantic equivalence. This results in Semantic Indistinguishability, where semantic signals are obscured by linguistic noise. Conversely, the T5-NSID matrix reveals a sharp block-diagonal structure. The dark diagonal squares represent cohesive synonymous sets, while the uniform background (${\hat{\bar{D}}}_{\mathrm{inter}}=0.9801$) indicates near-maximal separation between distinct synonymous sets. This high inter-synonymous set separability demonstrates that the model effectively filters out syntactic variations to isolate semantic characteristics.

**Figure 4:**
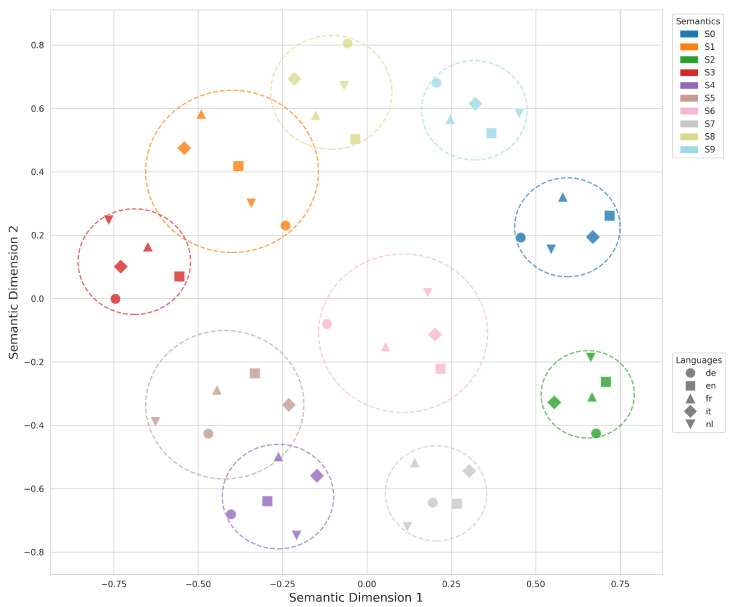
Two-dimensional MDS projection of the high-dimensional semantic space. Different colors and dashed circles indicate different synonymous sets, while marker shapes denote different languages.

*C. Geometric Projection:* To further validate this topological separation, we applied Multidimensional Scaling (MDS) to project the high-dimensional semantic distances into a 2D space, illustrated in Figure 4. The visualization yields N=10 clearly demarcated clusters, explicitly bounded by dashed circles. Each circle encapsulates a distinct cross-lingual synonymous set. Crucially, within each synonymous set, the different geometric shapes (representing the 5 distinct languages: English, French, German, Dutch, and Italian) are tightly clustered and intermixed. Meanwhile, the vast spatial separation between disparate circles confirms high inter-synonymous set separability. This geometric evidence visually supports that our metric successfully overcomes cross-lingual surface variation to capture language-invariant semantic equivalence.

For the global summary in Table 2, we aggregate the discrete estimators introduced in Section 5.3 over all *N* synonymous sets. Specifically, ${\hat{\bar{C}}}_{\mathrm{intra}}$ is obtained by averaging the estimated intra-synonymous set cohesion ${\hat{C}}_{\mathrm{intra}}\left(S_{i}\right)$ over all synonymous sets, while ${\hat{\bar{D}}}_{\mathrm{inter}}$ is obtained by averaging the estimated inter-synonymous set separability ${\hat{D}}_{\mathrm{inter}}\left(S_{i},S_{j}\right)$ over all unordered pairs of distinct synonymous sets. The reported global discrimination ratio ${\hat{\bar{R}}}_{\mathrm{disc}}$ is then computed as the ratio between these two aggregated quantities, thereby providing a corpus-level summary of the topological separation induced by each distance measure.

### 6.2. Microscopic Analysis: Semantic Friction and Synonymous Set Hardness

While the T5-NSID metric achieves high global separability, the intra-synonymous set cohesion is not absolute (${\hat{C}}_{\mathrm{intra}}>0$). Because the semantic concepts underlying these synonymous sets possess distinct linguistic characteristics, the structural variations within individual synonymous sets warrant a closer investigation. To understand the source of this residual distance, we decompose the intra-set distance of each experimental synonymous set $S_{i}$ into an intrinsic monolingual baseline $\epsilon_{\mathrm{mono},i}$, estimated from monolingual paraphrasing, and the observed cross-lingual cohesion cost $C_{i}:={\hat{C}}_{\mathrm{intra}}\left(S_{i}\right)$, derived from the off-diagonal pairwise T5-NSID distances among different expressions within the same synonymous set. Here, $\epsilon_{\mathrm{mono},i}$ reflects the system-level noise caused by monolingual paraphrasing, whereas $C_{i}$ captures the actual intra-set distance after cross-lingual semantic alignment. We formally define Semantic Friction as their difference: $\Delta_{i}=C_{i}-\epsilon_{\mathrm{mono},i}$.

**Figure 5:**
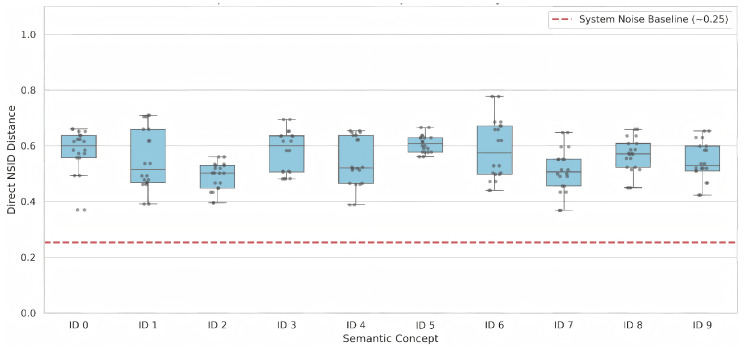
Micro-analysis of semantic friction across different cross-lingual synonymous sets. Gray dots denote individual off-diagonal pairwise NSID distances between different expressions within the same synonymous set, the boxplots summarize their distribution, and the red dashed line indicates the global monolingual noise baseline, ${\bar{\epsilon }}_{\mathrm{mono}}\approx 0.25$.

Figure 5 presents a micro-analysis of this phenomenon. The gap between the cross-lingual boxplots and the monolingual noise baseline effectively quantifies the “energy cost” or friction incurred when translating a meaning across different languages. Table 1 provides a granular breakdown of this friction across all synonymous sets, revealing distinct patterns of translation hardness. For instance, synonymous sets representing abstract concepts, such as S0 (Tech) and S6 (Legal/Order), exhibit high friction (Δ>0.36). Because these underlying meanings lack rigid physical referents, their structural representations undergo significant deformation when mapped across different linguistic rules. Conversely, S3 (Sports/Entity) demonstrates the lowest friction (Δ=0.215). Although paraphrasing specific entities like “Vidal” introduces high intrinsic noise ($\epsilon_{\mathrm{mono},3}=0.365$), the cross-lingual translation remains remarkably stable. Such entity-centric terms act as semantic anchors that strongly resist structural deformation during translation.

Ultimately, this micro-analysis demonstrates that cross-lingual semantic equivalence is not a rigid binary state, but rather a spectrum governed by the inherent translation hardness of the synonymous sets. By isolating and quantifying this semantic friction, the T5-NSID metric not only provides a highly discriminative measure for global semantic alignment, but also offers a practical, interpretive lens for understanding the difficulty of mapping complex meanings across diverse languages.

## 7. Conclusions

In this paper, we have established Semantic Algorithmic Information Theory to address the fundamental limitations of classical, syntax-bound algorithmic information theory. By integrating standard Turing machines with computable synonymous mappings, we formalized the Semantic Turing Machine System (STMS) and Semantic Complexity. This framework theoretically decouples the informational value of abstract concepts from the algorithmic cost of their diverse syntactic realizations. To transition from theory to computable metrics, we introduced a model-based Direct NSID estimator as a computable approximation to the ideal Normalized Semantic Information Distance using modern neural autoregressive estimators. Through a set-theoretic evaluation framework, we quantified the phenomenon of Semantic Equivalence—demonstrating that appropriate computational systems can suppress syntactic noise and preserve semantic structure more consistently across diverse surface realizations.

Looking forward, this framework provides a theoretical basis for several future research directions. Future work could systematically analyze the gap between syntactic and semantic entropy across diverse language families. Additionally, extending our metric from static objects to dynamic stochastic processes could offer new mathematical perspectives on cognitive modeling and language evolution. By elevating Kolmogorov complexity from syntactic descriptions toward conceptual representations, this work aims to contribute to the development of semantic-aware information metrics. 

## Figures and Tables

**Table 1:** Full quantification of semantic friction and synonymous set hardness. Values are reported as mean ± standard error.

Synonymous Set	Topic/Context	$\epsilon_{\mathrm{mono},i}$	$C_{i}={\hat{C}}_{\mathrm{intra}}\left(S_{i}\right)$	Friction ($\Delta_{i}$)	Hardness
S0	Stanford Chip (Med-Tech)	0.187 ± 0.01	0.577 ± 0.08	0.390	High
S1	Disease Detection (Health)	0.212 ± 0.02	0.552 ± 0.11	0.340	Medium
S2	Plane Crash (Incident)	0.230 ± 0.04	0.489 ± 0.05	0.259	Medium
S3	Vidal Football (Sports)	0.365 ± 0.06	0.580 ± 0.07	0.215	Low
S4	Protest Start (Politics)	0.282 ± 0.05	0.544 ± 0.09	0.262	Medium
S5	Traffic Block (Action)	0.335 ± 0.04	0.608 ± 0.03	0.274	Medium
S6	Police Request (Order)	0.225 ± 0.02	0.586 ± 0.11	0.361	High
S7	Protest Route (Location)	0.194 ± 0.02	0.511 ± 0.08	0.317	Medium
S8	Nadal Tennis (Sports)	0.283 ± 0.02	0.568 ± 0.06	0.285	Medium
S9	King of Clay (Quote)	0.220 ± 0.02	0.545 ± 0.07	0.325	Medium

**Table 2:** Global quantitative comparison of macroscopic metrics.

Metric	Gzip	Ours (T5)	Improvement
Intra-Synonymous Set Cohesion (${\hat{\bar{C}}}_{\mathrm{intra}}$)	0.6301	0.5560	+11.8%
Inter-Synonymous Set Separability (${\hat{\bar{D}}}_{\mathrm{inter}}$)	0.7547	0.9801	+29.9%
Discrimination Ratio (${\hat{\bar{R}}}_{\mathrm{disc}}$)	1.1977	1.7629	+47.2%

## Data Availability

The data that support the findings of this study are available from the corresponding author upon reasonable request.

## Abbreviations

The following abbreviations are used in this manuscript:

AIT	Algorithmic Information Theory
NCD	Normalized Compression Distance
NID	Normalized Information Distance
NSID	Normalized Semantic Information Distance
STMS	Semantic Turing Machine System

## Appendix A

This appendix presents a multilingual synonymous set example (ID: S6) from Section 6, with equivalent realizations in English, French, German, Dutch, and Italian.

**Table A1:** Multilingual synonymous set for S6 used in the experiments.

Language	Sentence
en	At 11:20, the police asked the protesters to move back on to the pavement, stating that they needed to balance the right to protest with the traffic building up.
fr	À 11 h 20, la police a demandé aux manifestants de retourner sur le trottoir, déclarant qu’ils devaient trouver un équilibre entre le droit de manifester et l’augmentation de la circulation.
de	Um 11:20 Uhr forderte die Polizei die Demonstranten dazu auf, wieder auf die Bürgersteige zurückzukehren, und gab dazu an, dass man das Demonstrationsrecht mit dem Verkehrsaufkommen abwägen müsse.
nl	Om 11:20 vroeg de politie aan de demonstranten om weer op de stoep te gaan staan, waarbij zij het recht aanhaalde om het recht op protest af te wegen tegen het toenemende verkeer.
it	Alle 11:20 la polizia ha chiesto ai manifestanti di spostarsi sul marciapiede richiamando l’esigenza di trovare un equilibrio tra il diritto a manifestare e il traffico in aumento.
